# Analysis of the physicochemical properties, cytotoxicity and volumetric changes of AH Plus, MTA Fillapex and TotalFill BC Sealer

**DOI:** 10.4317/jced.57527

**Published:** 2020-11-01

**Authors:** Marcela-Milanezi Almeida, Clarissa-Teles Rodrigues, Adriana-Arruda Matos, Kleber-Kildare-Teodoro Carvalho, Emmanuel-João-Nogueira-Leal Silva, Marco-Antonio-Hungaro Duarte, Rodrigo-Cardoso Oliveira, Norberti Bernardineli

**Affiliations:** 1 Department of Dentistry, Endodontics and Dental Materials, Bauru School of Dentistry, University of São Paulo, São Paulo, Brazil; 2 Department of Biological Sciences, Bauru School of Dentistry, University of São Paulo, Bauru, São Paulo, Brazil; 3 Department of Restorative Dentistry, School of Dentistry of Ribeirão Preto, University of São Paulo, Ribeirão Preto, São Paulo, Brazil; 4 Department of Endodontics, School of Dentistry, Grande Rio University (UNIGRANRIO), Rio de Janeiro, Brazil

## Abstract

**Background:**

To evaluate the physicochemical properties and cytotoxicity of AH Plus, MTA Fillapex and TotalFill BC Sealer. Volumetric changes were also evaluating using micro-computed tomography (micro-CT).

**Material and Methods:**

Radiopacity and flow were evaluated in accordance with the ISO 6876, while setting time was evaluated in accordance with the ASTM- C266-08 specifications. The release of Ca2+ ions and pH were measured with spectrophotometer and pH meter, respectively, after different time intervals (1h, 3h, 24h, 72h, 168h, and 360h). Cytotoxicity was evaluated by MTT reduction assay to check 3T3 cells viability at 24, 48 and 72 hours. Volumetric change was evaluated by micro-CT, by using 30 acrylic teeth, filled with gutta-percha cones and the tested root canal sealer. The samples were evaluated after 168h, 360h and 720h of immersion in distilled water. Data were statistically analyzed by one-way ANOVA and Tukey test or by Kruskal-Wallis and Dunn tests (*P*<0.05).

**Results:**

MTA Fillapex and TotalFill BC Sealer showed lower radiopacity than AH Plus (*P*<0.05). The MTA Fillapex showed the highest flow, while AH Plus showed the lowest flow (*P*<0.05). The initial and final setting time of AH Plus were lower than MTA Fillapex and TotalFill BC Sealer (*P*<0.05). In general, TotalFill BC Sealer presented higher Ca2+ ion release and pH than the other tested sealers. TotalFill BC Sealer also showed overall lower cytotoxicity when compared to the other sealers. Volumetric change of AH Plus and TotalFill BC Sealer was lower than MTA Fillapex (*P*<0.05).

**Conclusions:**

AH Plus, MTA Fillapex and TotalFill BC Sealer showed slight differences in the physicochemical properties and cytotoxicity, but all suitable for an endodontic sealer. However, AH Plus and TotalFill BC Sealer showed low volumetric changes when compared to MTA Fillapex.

** Key words:**Calcium silicate, cytotoxicity, physicochemical properties, micro computed tomography.

## Introduction

The infiltration of irritating substances and microorganisms in the periapical tissues is responsible for most of the endodontic failures (1,2). Therefore, an ideal filling material should offer specific properties, such as tissue tolerance, ideal setting and working time, adhesiveness, radiopacity, bacteriostatic properties, solubility in solvents and insolubility in oral and tissue fluids (3). Moreover, in cases of leakage of root canal filling material into the periapical tissues, it is important for the material to be as much as compatible as possible, reducing toxicity and inflammatory action in these tissues (4).

Root canal sealers are classified according to their main components, such as resin, calcium hydroxide, silicon, calcium silicate and others (3). AH Plus Dentsply De Trey Gmbh, Konstanz, Germany), an epoxy resin-based sealer, is considered the gold standard of endodontic sealers because of its excellent physicochemical properties (5,6), despite not showing bioactive potential (7-10). Hydrophilic calcium-silicate based root canal sealers, such as MTA Fillapex (Angelus, Londrina, Brazil) and Totalfill BC Sealer (FKG Dentaire SA, La Chaux-de-Fonds, Switzerland) instantly attracted the dental community, because some of them were premixed, injecTable, hydrophilic, and bioactive root-filling materials. Overall, the body of evidence made available over the last few years has shown that such hydrophilic calcium-silicate root canal sealers are biocompatible and bioactive, features mostly attributed to the presence of calcium phosphate in their composition (8-10). Moreover, it seems that this class of sealers can interact with the surrounding dentinal tissue by its ability to form hydroxyapatite, thereby establishing a real connection between the filling material and dentin (11). However, other critical physicochemical properties, such as solubility, are still under scientific scrutiny.

Low solubility is one of the most desirable physical properties of an endodontic sealer, as it can greatly influence the endodontic treatment success. More soluble materials can release irritating substances and increase the risk of dispersion and colonization of bacteria in the periapical region. Commonly, the solubility of root canal sealers are tested experimentally, based on methods described by the International Organization of Standardization (ISO 6876:2001) (12), by the American National Standards Institute / American Dental Association (ANSI/ADA 57:2000) (13) or by the American Society for Testing and Materials (ASTM C266-07:2007) (14). A major problem with these *in vitro* experimental models is that it does not simulate clinical conditions. For these limitations, previous studies recommended the use of micro-CT technology and simulated root canal treatment to evaluate the volumetric changes of root canal sealers after immersion of these filled samples in distilled water or simulated body fluid (15). However, such information is limited in the Endodontic literature.

Therefore, the aim of the present study was to analyze the radiopacity, setting time, flow, pH, calcium ion release and cytotoxicity of AH Plus, Total Fill BC Sealer and MTA Fillapex. Moreover, the volumetric changes were also evaluated using micro-CT technology. The null hypotheses tested were that.

(i) There are no differences in the radiopacity, setting time, flow, pH, calcium ion release and cytotoxicity of the tested root canal sealers.

(ii) There are no differences in the volumetric changes of the tested root canal sealers.

## Material and Methods

Three sealers were used in the present study: TotalFill BC Sealer (FKG Dentaire SA, La Chaux-de-Fonds, Switzerland), MTA Fillapex (Angelus, Londrina, Brazil) and AH Plus (Dentsply De Trey Gmbh, Konstanz, Germany). Preparation and handling of the sealers was carried out in accordance with manufacturer’s instructions.

-Radiopacity 

Nine cylindric samples of each material were manufactured by pouring the manipulated root canal sealers into metallic rings with an internal diameter of 10 mm, and 1 mm thick. The filled rings were kept at 37° C until the sealers were completely set. Then, the specimen thicknesses were confirmed with a Digital caliper (Mitutoyo Corp., Tokyo, Japan). The root canal sealer specimens, a 1 mm thick dentin block (used as a control) and an aluminum scale (graded from 2 to 16 mm Al) were radiographed on occlusal films (F-velocity; Kodak Comp, Rochester, NY, USA), within the following parameters: 60 kV and 10 mA for 0.3 seconds (Gnatus XR 6010; Gnatus, Ribeirão Preto, Brazil), with a focus-film distance of 30 cm. After processing, the radiographs were scanned with a digital scanner and imported into the Digora 1.51 software (Orion Corporation Soredex, Helsinki, Finland). The radiographic density data were converted into mmAl, as described in a previous study (16).

-Flowability 

A volume of 0.5 mL of sealer was placed on a glass plate in accordance with ISO 6875 specifications (12). Three minutes after starting the spatulation, another plate with a mass of 20 ± 2 g and a load of 100 g plus was applied centrally on top of the plate. Ten minutes after the start of mixing, the load was removed, and the average of the major and minor diameters of the compressed sealer was measured using a digital caliper. Three measurements were performed for each sealer.

-Setting time 

The test was conducted under controlled temperature and humidity conditions of 37 ± 1°C and 95% ± 5%, respectively. For the setting time, nine metal rings (n = 3) with 10 mm diameter and 2 mm thickness were filled with manipulated root canal sealer, according to the American Society for Testing and Materials Specifications (ASTM- C266-08) (ref). After 180 seconds, a Gilmore 113.4g needle was used in each specimen at 60 second intervals, according to the ISO 6876: 2001 specification (12). Once it was not possible to check any mark on the sample surface, the initial setting time was established. A Gilmore 453.6g needle was used in the same manner to determine the final set time. Three specimens were evaluated for each group.

-pH level and calcium release 

Thirty acrylic teeth (10 per group) were used, which were previously prepared with Flex-Gold rotary instruments (Easy Equipamentos Odontológicos, Belo Horizonte, Brazil) up to instrument 30/0.09 and then used filled with the gutta-percha ProTaper F3 (Dentsply-Sirona, Baillagues, Switzerland) and the tested sealers. The coronal portion was sealed with Bioplic material (Biodinâmica Química e Farmacêutica, Ibiporã, Brazil). The specimens were individually placed in glass vials containing 10 mL of ultrapure water (Milli-Q water; Purelab, Analítica, Brazil) and stored at 37° C where they remained throughout the experimental period. To avoid any interference in the results, all glass vials were pre-treated with nitric acid. The levels of pH and calcium release readouts were performed at time intervals of 1, 3, 24, 72, 168 and 360 hours. After each experimental period, the teeth were transferred to a new flask with the same volume of ultrapure water.

The pH was measured with a pH meter (model 371: Micronal, São Paulo, Brazil), previously calibrated using controls with pH values 4, 7 and 14. After the specimens were removed, the containers were placed in an agitator (Model 251; Farmem, São Paulo, Brazil) for 5 seconds before each measurement. Deionized water was used as a control for the pH level measurements in all time intervals analyzed.

Calcium ion release was evaluated by means of an atomic absorption spectrophotometer (AA6800; Schimadzu, Tokyo, Japan) equipped with a calcium ion-specific hollow cathode lamp. All samples were analyzed at the same time as pH level analyses were performed. To avoid possible alkali metal interference, a lanthanum solution was prepared by diluting 9.8 g of lanthanum nitrate in 250 mL of acid solution. A stock solution of calcium was prepared by diluting 2.4972 g of calcium carbonate in 50 mL of ultrapure water. To this solution, 10 mL of concentrated hydrochloric acid was added, diluted with 1000 mL of ultrapure water, so that 1 mL of this solution corresponded to 1 mg of calcium. From this solution, calcium solutions were prepared in the following concentrations: 20 mg/L-1; 10 mg/L-1; 5 mg/L-1; 2.5 mg/L-1; 1.25 mg/L-1; and 2 mL of the lanthanum nitrate solution was added to 6 mL of calcium or test solution. To prepare the blank, 6 mL of ultrapure water was added to the same amount of the lanthanum nitrate solution. Calcium ion release readouts were compared with a standard curve obtained from standard solution readouts.

-Cytotoxicity 

The initial concentration used for the experiment was 100 mg of each root canal sealer in 1 mL DMEM (Dulbecco’s Modified Eagle’s Medium - Sigma-Aldrich, St. Louis, MO, USA) supplemented with 10% FBS (Fetal Bovine Serum - Gibco). The sealers conditioned medium was kept overnight at 37° C, under sterile conditions, with 5% CO2 for 24 hours. After these steps, serial dilutions of the media conditioned with the sealers were performed, in accordance with the ISO 10993-5 recommendations (17). The concentrations used were 50 mg/mL, 10 mg/mL, 5 mg/mL, 1 mg/mL and 0 mg/mL (negative control group).

*In vitro* cytotoxicity of the sealers was evaluated by using NIH3T3 murine fibroblasts from the ATCC-American Type Culture Collection. These fibroblasts were cultured in DMEM culture medium 10% FBS and incubated at 37°C containing 5% CO2. After reaching subconfluence, the cells were subcultured using the trypsin enzyme (0.25% porcine trypsin (1:250) in HBSS, with 0.1% EDTA- Sigma-Aldrich), and the cell count was performed by using trypan blue dye. Subsequently, the cells were used for cytotoxicity assays.

For viability assays 2x103 cells/well were plated in 96-well plates. After incubation for 24 hours, the wells were refreshed with the culture media conditioned with sealers, in addition to the negative control (DMEM 10% SFB). The MTT (3- (4,5- dimethylthiazol-2-yl) -2,5-diphenyltetrazolium bromide) (Sigma-Aldrich) reduction assays were performed according to Mosmann (18). In each experimental time-point (24, 48 and 72 hours) the wells were washed with 1X PBS (Phosphate buffered saline), then the cells were incubated in a 1 mg MTT solution to 1 mL DMEM without FBS. After these procedures, the plates were left at 37º C for 4 hours; then the solution was removed, the pigment was extracted with DMSO (Dimethylsulfoxide- Synth, Labsynth, São Paulo, Brazil) and left at room temperature for 30 minutes. The absorbance was measured in spectrophotometer at 562 nm (Synergy TM Monochrome-Based Multi-Mode Microplate Reader, BioTek Instruments Inc, Winooski, Vermont, USA) (ref). All assays were repeated in triplicate.

-Volumetric change

Volumetric change was determined by micro-CT images (15). Each specimen was scanned four times. Thirty acrylic central incisors (n = 10) were used, which were previously prepared with Flex-Gold rotary instruments (Easy Equipamentos Odontológicos) up to instrument 30/0.09. Samples were filled up to the working length with single cone F3 ProTaper. Subsequently, the samples were scanned with a desktop X-ray microfocus CT scanner (SkyScan 1174v2; SkyScan, Kontich, Belgium). The images were digitized by using a 50 kV X-ray tube voltage, 800 A anode currrent. The following image capture parameters were used: voxel size of 14.1 m with 1.1° rotation step using a 360° rotation. Each scan generated 327.tif images with 1024 X 1304 pixels. Digital data were compiled by reconstruction software (NRec-onv1.6.4.8. SkyScan), and the CTAN software (CTAN v1.11.10.0, SkyScan) was used for volume measurements. The region of interest (ROI) was delimited for each sample and these images were binarized. Quantitative analysis of material volume (mm3) was obtained. After this initial scanning process, each sample was individually immersed in a glass vial containing 15 mL of ultrapure water, and then stored at 37° C for time intervals of 168, 360 and 720 hours. After each experimental period the samples were scanned and analyzed again using exactly the same parameters as those set up for the first examination. Volumetric change of the samples from each group was determined by calculating the volume that was lost during immersion in ultrapure water, and the results found were converted to percentages to show the proportion of the material that was dissolved.

-Statistical analysis 

Data were analyzed for normality by the Kolmogorov-Smirnov test. The values of radiopacity, setting time, flow and cytotoxicity were compared by means of the ANOVA and Tukey tests (*P*<0.05). For the pH, calcium ion release and volumetric change the Kruskal-Wallis test was used and the post hoc analysis was performed using the Dunn test, for the multiple comparisons (*P*<0.05). Prism 5.0 Software (GraphPad Software Inc, La Jolla, CA, USA) was used as an analytical tool.

## Results

-Radiopacity 

The radiopacity values of all the sealers were higher than those recommended by ISO 6876/2001 (12). The AH Plus presented the highest radiopacity, while no statistically significantly differences were observed between TotalFill BC Sealer and MTA Fillapex (*P*<0.05) (Table 1).

**Table 1:**
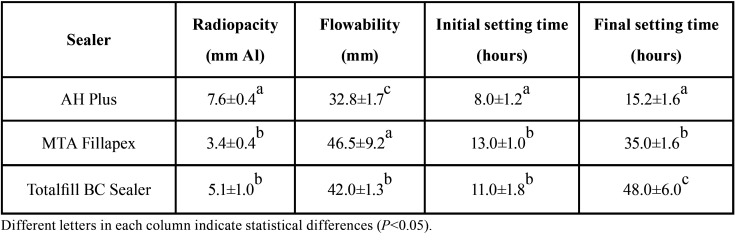
Mean and standard deviation of the radiopacity (mm Al), flow (mm), initial setting time (hours) and final setting time (hours).

-Flowability 

All tested sealers had a flow rate higher than 30 mm, which was in accordance with ISO 6876/2001 recommendations (ref). The MTA Fillapex showed the highest flow, while AH Plus showed the lowest flow (*P*<0.05) (Table 1).

-Setting time 

AH Plus showed the shortest initial and final setting time when compared to MTA Fillapex and Total Fill BC Sealer (*P*<0.05). While no differences between MTA Fillapex and TotalFill BC Sealer were observed in the initial setting time (*P*>0.05), the final setting time of TotalFill BC Sealer was lower than those of MTA Fillapex (*P* <0.05) (Table 1).

-pH

In general, pH analysis showed the lowest values for AH Plus, followed by MTA Fillapex and Totalfill BC Sealer. Slight variations of pH values were observed among the different tested time-points. These results can be visualized in Table 2.

**Table 2:**
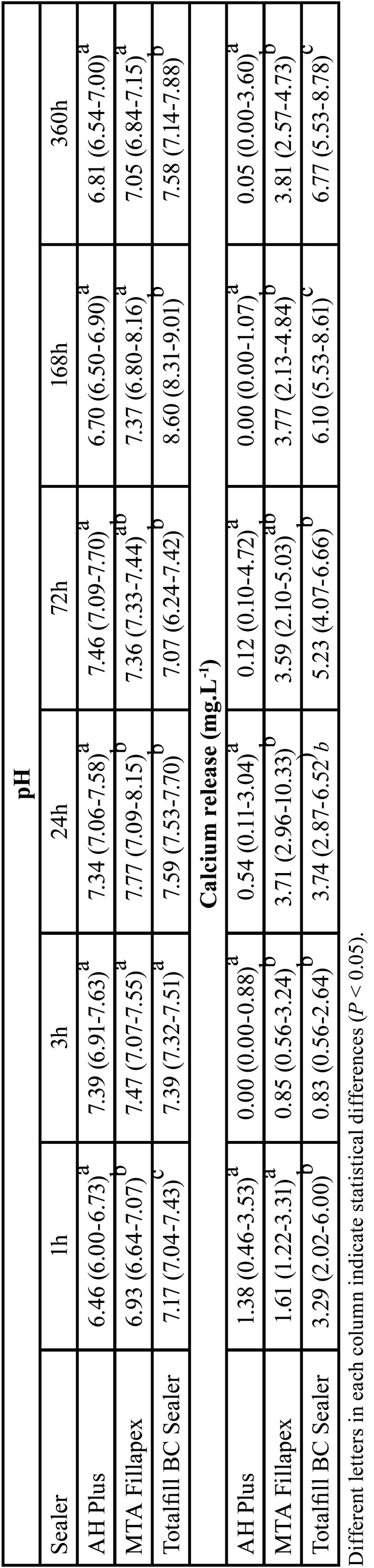
Median, minimum and maximum values of the cumulative pH and calcium release values (mg.L-1) through the different time points.

-Calcium release

In general, TotalFill BC Sealer was associated with the higher release of calcium ions in all tested time points. In contrast, AH Plus was associated with the lowest release of calcium ions. Calcium ion release increase in the TotalFill BC Sealer and MTA Fillapex along the time. These results can be visualized in Table 2.

-Cytotoxicity

Cytotoxicity of all tested root canal sealers was dose and time-point dependent. TotalFill BC Sealer generally showed the lower cytotoxicity values comparable to those obtained by the negative control group. In contrast, AH Plus and MTA Fillapex showed higher cytotoxicity than TotalFill BC Sealer with a trend of decreasing cytotoxicity with increasing contact time. These results can be observed in Table 3.

**Table 3:**
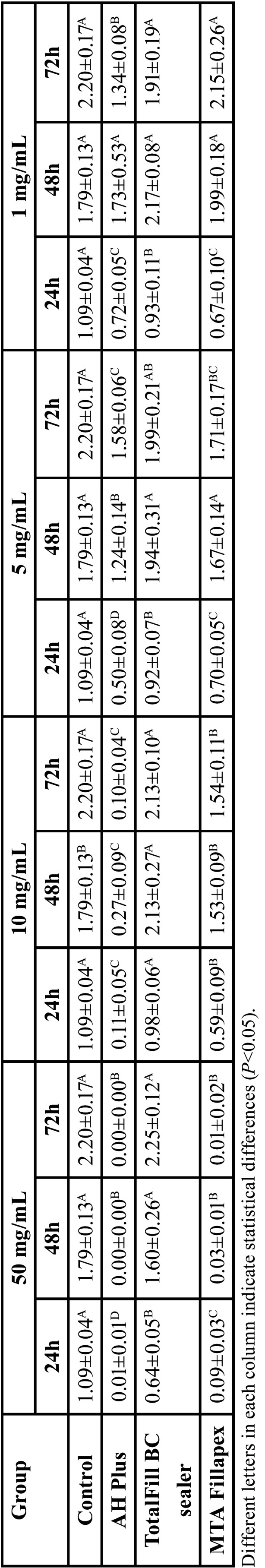
Mean and standard deviation of the cytotoxicity (absorbance) of the different root canal sealers, in different time points and concentrations.

-Volumetric changes

The volumetric change values of Totalfill BC Sealer and AH Plus were lower than those of MTA Fillapex in all tested time points (*P*<0.05). No differences were observed between TotalFill BC Sealer and AH Plus (*P*>0.05) (Table 4).

**Table 4:**
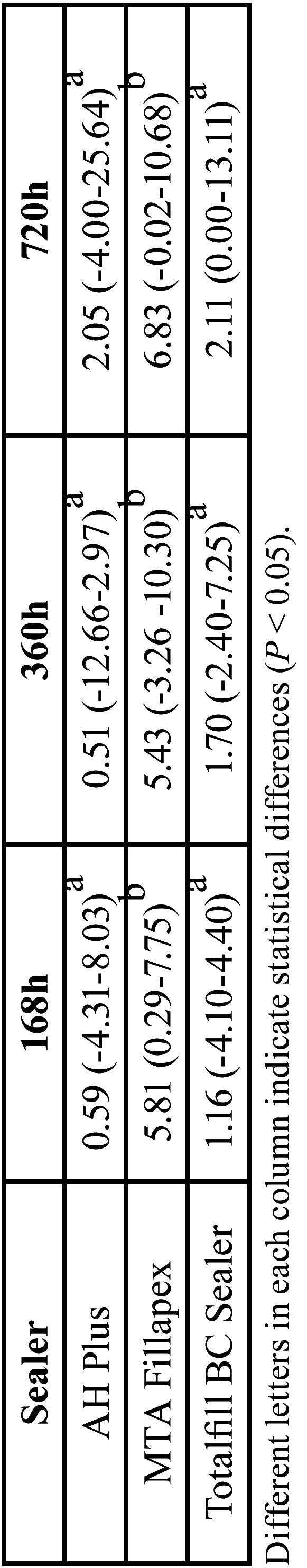
Median, minimum and maximum values of the cumulative volumetric change (%) of the tested groups through the different time points.

## Discussion

The present study evaluated the radiopacity, flow, setting time, pH, calcium ion release, cytotoxicity and the volumetric changes of AH Plus, Total Fill BC Sealer and MTA Fillapex. The radiopacity results demonstrated that all tested root canal sealers complied with the ISO 6876/2001 specification (12), that established the minimum radiopacity value of 3.00 mm Al. However, differences were observed among the tested root canal sealers, with AH Plus demonstrating the higher radiopacity when compared to MTA Fillapex and TotalFill BC Sealer. These differences might be explained by differences in the proportion and/or the type of radiopacifier present in the root canal sealer. The flowability of all tested root canal sealers was also in accordance with ISO 6876/2001 (12) minimum requirements of 20 mm. As the radiopacity, root canal sealers demonstrated differences in the flowability with MTA Fillapex demonstrating the higher flow values and AH Plus the lower one. In fact, these results are in accordance with a previously published study (5), and might be explained by the different composition of the tested sealers as well as differences in the size of the sealer particles – the smaller the particles, the greater the flow ability of the sealer.

The initial and final setting time was determined by following the ASTM-C266-08 recommendations (14). The AH Plus presented initial and final setting times of 8 and 15 hours, respectively, which is in agreement with the findings of previous studies (5,6). AH Plus is a paste-to-paste material that has an amine polymerization reaction contained in the epoxy resin. Totalfill BC Sealer is a premix of inorganic components and radiopacifiers that are premixed with a water-free liquid carrier; water is required for it to reach its final setting time. On the other hand, MTA Fillapex is a paste-to-paste material, and when the two pastes come into contact, this promotes two chemical reactions that are responsible for the setting of the material. Setting time of AH Plus was in accordance with the values provided by the manufacturer. In contrast, setting time of MTA Fillapex and TotalFill BC Sealer was more than 10 times longer than those values provided by the manufacturer´s. In fact, this discrepancy between the values provided by the manufacturers and those obtained in the present study, associated with data from other studies that showed that these materials were not able to set even under controlled conditions in tests lead us to reflect if these materials are really capable of setting in a predicTable way *in vivo*. -Future studies should be performed within this issue.

An alkaline pH has a destructive effect on bacterial cell membranes and protein structure (19). Moreover, a high pH may also neutralize the acids secreted by osteoclasts preventing further destruction of mineralized tissue (20). Also, high pH activates alkaline phosphatase involved in the mineralization process (20,21). In the present study, TotalFill BC Sealer and MTA Fillapex showed higher pH values than AH Plus. This difference reflected in the bioceramic particles present, in different concentrations, in both sealers. One disadvantage of this alkaline pH is a possible high cytotoxicity, which causes denaturation of adjacent cells (22). However, even with the high pH, TotalFill BC sealer was not associated with strong cytotoxicity effects, which will be discussed further. In addition to pH, the mechanism of stimulation of periapical repair by deposition of mineralized tissue also depends on Ca2+ release capacity (20,21). Moisture facilitates calcium silicate hydration reactions. The calcium and hydroxyl ion release from sealers that contain calcium silicate, results in the formation of an apatite layer that chemically binds to calcium silicate sealer and the dentin wall (9-11). The highest amount of Ca2+ release for Totalfill BC Sealer occurred in the time interval of 360 hours (6.77 mg/L) showing higher values than those of the other sealers. This factor may be related to the final setting time of this material, longer than the other tested materials. Further studies should be conducted to confirm this observation.

As physical-chemical properties, biological characteristics are essential for clinical success. The cytotoxicity of sealers can cause cell degeneration and delayed healing due to direct contact of the sealers with the periapical tissues, making a satisfactory periapical repair difficult. Bioceramic materials are considered promising for this repair, due to their excellent physicochemical and biocompatibility propertie. In the present study, sealers were in indirect contact with the cells, by eluting root canal sealer in DMEM. This methodology was closer to clinical since sealer will not preferentially come into contact with the cells. Tissue fluids or blood will come into contact with the sealer and carry on components to nearby cells. The results of the present study revealed minor or nonexistent cytotoxic effects for TotalFill BC Sealer. This sealer exhibited good biocompatibility with NIH3T3 cells, as well as a lower cytotoxic potential compared with AH Plus and MTA Fillapex. On the other hand, This study showed that AH Plus and MTA Fillapex showed higher cytotoxicity at high concentrations (50 mg / mL) than Totalfill BC Sealer in all time-points. This may have been caused mainly by small amounts of salicylate resin, diluting resin, and silica in MTA Fillapex or by release of the amine and epoxy resin components of AH Plus. Previous studies also showed the cytotoxic results of AH Plus and MTA Fillapex (5,22,23). Silva *et al.* (5) showed that AH Plus and MTA Fillapex was cytotoxic in fresh conditions and that AH Plus become noncytotoxic after 2 weeks while MTA Fillapex remained severely cytotoxic even after 4 weeks. Similar results were also obtained by Silva *et al.* (22) using a multiparametric cell viability assay. The results also demonstrated that after 1 week, AH Plus became noncytotoxic while MTA Fillapex remained severely cytotoxic over the entire experimental period on all evaluated parameters: XTT, neutral red and crystal violet dye elution. In fact, several other studies demonstrated that both sealers might be cytotoxic in different levels, depending on the test used, the cell line tested and the way the sealer was exposed. Therefore, it can be speculated that according to the amount of material extruding to the periapical tissues these materials can lead to damage in repair.

Sealer solubility can cause the release of chemical compounds that may irritate the periapical tissues and produce spaces in the filling mass inside the root canals, resulting in bacterial infiltration and proliferation. The solubility test usually follows technical standards provided by ANSI/ADA or ISO specifications. These methods consist of basic benchtop tests, immersion sealer samples in distilled water for a determined time-point and calculating weight loss after immersion. However, these *in vitro* models have the limitation that their results does not reflect directly the clinical condition. First of all, the standard methods recommend immersion of the materials in water only after complete setting, which is impossible to be achieved clinically because the materials are immediately placed into contact with fluids and blood. Moreover, clinically sealers are placed into root canals with gutta-percha points and only the main apical portion of root canal filling are in contact with perirradicular tissue. Therefore, the solubility values in a clinical scenario are probably different than the ones found in *in vitro* tests. Considering these limitations and not invalidating the technical standards recommendations, in the present study micro-CT technology to evaluate volumetric changes in a simulated clinical scenario. In this method, teeth were prepared and filled just as in clinical situation and then immersed in distilled water for 168, 360 and 720 hours. After these time points teeth were rescanned and evaluated regarding the volumetric changes. According to the present methodology, no differences were observed between TotalFill BC sealer and AH Plus (both lower than 3%), while MTA Fillapex showed the higher values of volumetric change. It is well known by previous studies using ANSI/ADA or ISO specifications that AH Plus has low solubility while MTA Fillapex has a high solubility (5,6,25). This is in accordance with the present results. TotalFill BC Sealer presented contrasting results in the literature: while some studies demonstrated low solubility (26,27) other demonstrated a high solubility.

## Conclusions

AH Plus, MTA Fillapex and TotalFill BC Sealer showed slight differences in the physicochemical properties and cytotoxicity, but all suitable for an endodontic sealer. However, AH Plus and TotalFill BC Sealer showed low volumetric changes when compared to MTA Fillapex.
